# The Role of Oxygen Free Radicals in Acute Renal Failure Complicating Obstructive Jaundice: An Experimental Study

**DOI:** 10.1155/1998/47363

**Published:** 1998

**Authors:** Serdar Yüceyar, Koray Gümüştaş, Süphan Ertürk, Ismail H. Hamzaoğlu, Nesrin Uygun, Meltem Ayaz, Ali Cengiz, Yilmaz Kafadar

**Affiliations:** 1 Department of Surgery Cerrahpaşa Medical Faculty University of Istanbul Turkey; 2 Department of Biochemistry Cerrahpaşa Medical Faculty University of Istanbul Turkey; 3 Department of Pathology Cerrahpaşa Medical Faculty University of Istanbul Turkey; 4 Department of Nephrology Cerrahpaşa Medical Faculty University of Istanbul Turkey

## Abstract

Oxydant injury is considered to be an important mechanism in the pathophysiology of acute renal failure. It has been thought that decrease in extracellular and intracellular fluid and endotoxemia seen in obstructive jaundice may cause an increase in production of oxygen free radicals and impairment in antioxydant defense mechanism. This study is designed to investigate the possible role of oxydant injury in renal failure seen in jaundiced patients. In this study, 28 rats were divided into four groups: Control(C) (N=7); Renal ischemia (RI) (N=7); Obstructive jaundice+renal ischemia (OJ+RI) (N=7); Obstructive jaundice (OJ) (N=7). All groups were compared with each other according to renal failure findings and enzyme activities, such as Xanthine oxidase (XOD), Superoxide Dismutase (SOD) and Catalase in renal cortex and Glutathione Peroxidase (GSH-Px), in blood at 3rd day after ischemia and reperfusion. Renal failure findings monitored by blood urea and creatinine levels, seemed more evident in OJ+RI than RI group (*p* <0.05). When compared with RI, in OJ+RI group, increase in XOD activity at 3rd day was statistically significant [0.259 ±0.01 U/g (tissue) and 0.362±0.03 U/g (tissue) respectively] (*p* <0.05). SOD and GSH-Px activities of each ischemic group at 3rd day were decreased compared to non-ischemic groups. This fall was significant (*p* <0.05). But there was no statistical difference between jaundiced and non-jaundiced groups. Alterations in catalase activities also had no statistical significance.

These findings may suggest that the injury induced by oxygen free radicals at re-oxygenation of tissue after ischemia may also play a role in the pathogenesis of acute renal failure developed in obstructive jaundice.


*HPB Surgery*, 1998, Vol. 10, pp. 387–393
Reprints available directly from the publisher
Photocopying permitted by license only

© 1998 OPA (Overseas Publishers Association)
Amsterdam B.V. Published under license
under the Harwood Academic Publishers imprint,
part of The Gordon and Breach Publishing Group.
Printed in India.

### The Role of Oxygen Free Radicals in Acute Renal Failure Complicating Obstructive Jaundice: An Experimental Study

SERDAR YÜCEYAR[a,*], KORAY GÜMÜŞTAŞ[b], SÜPHAN ERTÜRK[a], ISMAIL H. HAMZAOĞLU[a], NESRIN UYGUN[c], MELTEM AYAZ[d], ALI CENGIZ[a] and YILMAZ KAFADAR[a]

[a]*Department of Surgery,* [b]*Department of Biochemistry,* [c]*Department of Pathology,* [d]*Department of Nephrology, Cerrahpaşa Medical Faculty, University of Istanbul, Turkiye*

*(Received 30 July 1995; In final form 10 February 1997)*

**Oxydant injury is considered to be an important mechanism in the pathophysiology of acute renal failure. It has been thought that decrease in extracellular and intracellular fluid and endotoxemia seen in obstructive jaundice may cause an increase in production of oxygen free radicals and impairment in antioxydant defense mechanism. This study is designed to investigate the possible role of oxydant injury in renal failure seen in jaundiced patients. In this study, 28 rats were divided into four groups: Control(C) (N=7); Renal ischemia (RI) (N=7); Obstructive jaundice+renal ischemia (OJ+RI) (N=7); Obstructive jaundice (OJ) (N=7). All groups were compared with each other according to renal failure findings and enzyme activities, such as Xanthine oxidase (XOD), Superoxide Dismutase (SOD) and Catalase in renal cortex and Glutathione Peroxidase (GSH-Px), in blood at 3rd day after ischemia and reperfusion. Renal failure findings monitored by blood urea and creatinine levels, seemed more evident in OJ+RI than RI group ($p < 0.05$). When compared with RI, in OJ+RI group, increase in XOD activity at 3rd day was statistically significant [0.259 $\pm$0.01 U/g (tissue) and 0.362$\pm$0.03 U/g (tissue) respectively] ($p < 0.05$). SOD and GSH-Px activities of each ischemic group at 3rd day were decreased compared to non-ischemic groups. This fall was significant ($p < 0.05$). But there was no statistical difference between jaundiced and non-jaundiced groups. Alterations in catalase activities also had no statistical significance.**

**These findings may suggest that the injury induced by oxygen free radicals at re-oxygenation of tissue after ischemia may also play a role in the pathogenesis of acute renal failure developed in obstructive jaundice.**

*Keywords:* Obstructive jaundice, acute renal failure, oxygen free radical, Xanthine Oxidase, Superoxide Dismutase, Catalase, Glutathione Peroxidase

### INTRODUCTION

Acute renal failure (ARF) developed after obstructive jaundice continues to be a significant challenge, involving 6–18% of patients and associated with high mortality (25–100 %) [1, 2].

Ethiological factors of ARF seen in extrahepatic cholestasis are as follows; nephrotoxicity, haemodynamic changes, endotoxemia, enhanced prostaglandins and renal vascular reactivity

* Correspondence to: SERDAR YÜCEYAR, Turunçlu sok., Uğur apt., No: 23/A, D:25, Merter, 34010 Istanbul–Turkey.

[1–7]. In this respect, bile salts [8], lactulose [8], antibiotics [2], inhibition of production of prostaglandins [4, 9], the preoperative drainage of bile [2], mannitol [10], and dopamine [11] have been used for the prevention of ARF in various clinical and experimental studies.

It has been shown that oxygen free radicals play an important role in the pathophysiology of ARF developing after renal ischemia in experimental models [12–16]. We thought that the oxidants may also play a role in the development of renal failure in jaundiced rats by the stimulating effect of endotoxin through the lipoxygenase pathway of arachidonic acid metabolism [17, 18], the decrease of antioxydant enzyme activities in volume depletion [16], the disturbances in body-water compartments in jaundiced patients [10], the increases of reactive oxygen species in jaundice [19, 20], and the role of decrease of antioxidant defenses in liver injury in jaundiced rats [21]. We aimed to design this study to compare the activities of antioxidant enzymes [superoxide dismutase (SOD) and catalase (CAT)] in the renal cortical tissue and Glutathione peroxidase (GSH-Px) in whole-blood and xanthine oxidase in renal cortical tissue following renal ischemia/reperfusion in jaundiced and non-jaundiced rats.

### MATERIAL AND METHODS

Twenty-eight Wistar-Albino rats, weighing 190–225 g, were selected for the study. They were fed with free access to an unrestricted standard laboratory diet and water.

The rats were divided into four groups as follows: Control (C) ($n$=7 rats), Renal ischemia/reperfusion (RI) ($n$=7 rats), Obstructive jaundice+Renal ischemia/reperfusion (OJ+RI) ($n$=7 rats) and Obstructive jaundice (OJ) ($n$=7 rats). The rats which died during or after the manipulations were excluded from the study and new rats were operated on to equalize the number of rats of each group. Diethyl-ether was used for anesthesia in all groups. Vertical midline incisions were performed and the common bile duct was ligated and divided at the extrapancreatic portion of the duct to create the jaundiced group ($n$=14 rats). Laparotomies were done in 7 randomly selected rats from the jaundiced group on day 7 and 7 non-jaundiced rats. Left and right renal pedicles were clamped for 30 minutes to create complete renal ischemia. The clamps were then removed and the abdominal incision was closed. The group OJ on day 7, the group C and the groups of RI and OJ+RI three days after the ischemia were sacrified by taking whole body blood from the heart by puncture under general anesthesia. The blood samples were taken into standard heparin solution (1/1) for the activity of glutathione peroxidase. The left and right kidneys of each rat were removed in all groups and kept with iced 0,9 % NaCl solution for short time. A portion of dimension 0,5 × 0,5 cm of kidneys (left or right) which contain both renal cortical and medullar tissue were fixed in 10 % solution of formaline for histological evaluation. The rest of the kidney tissues were used for the measurement of the enzyme activities.

### MEASUREMENT OF ENZYME ACTIVITIES

The kidney cortical tissue was weighed and homogenized with a Teflon-potter homogenizer in 0,1 mol/L Tris HCL, at pH 8,1 (20 % w/v), at 4°C. The homogenate was sonicated and centrifuged at 100.000 × g for 30 minutes, and the supernatant was removed and stored frozen (−20°C) for at least 24 hours before the enzyme assay. The amount of xanthine oxidase was determined by measuring the rate at which uric acid was formed at 37°C [22]. Superoxide dismutase was determined by the nitroblue tetrazolium (NBT) method [23] Catalase and glutathione peroxidase activities were measured by using the methods of Beers [24] and Paglia [25] respectively.

### HISTOLOGIC EVALUATION

Following fixation in 10% formaline solution, the samples were embedded in paraffin; Cross-sections, 3 to 4 μm thick, were stained with Hematoxylin and Eosin. Changes found in acute renal failure were scored semiquantitatively. A minimum of 100 cortical tubular profiles from at least 10 different regions of kidney were assessed with a 40*x* objective and scored for each tubulus. The absence (0) or presence (1) of tubular cytoplasmic vacuolisation, cell membrane blebbing (1 or 2), tubular epithelial cell flattening (1), brush border loss (1), interstitial edema (1), cell necrosis (1 or 2), and tubular lumen obstruction (1 or 2) was evaluated and an average score derived for each specimen and group [16]. The results of groups were crossed.

### STATISTICAL ANALYSIS

For comparison among groups, one way analysis of variance (ANOVA) and Turkey's-HSD test were performed [26]. All values are expressed as the mean ±SD. $p < 0,05$ was considered to be statistically significant.

### RESULTS

The bilirubin levels were significantly higher in the bile duct ligated groups than the groups with unligated bile duct ($p < 0,05$). The blood urea and creatinine levels were higher in the OJ+RI and RI groups than non-ischemic groups ($p < 0,05$) and when compared with RI, a significant difference was also observed in OJ+RI ($p < 0,05$). In histologic evaluation, the differences were only found in tubular cytoplasmic vacuolisation and brush border loss. Scoring results showed that values of OJ+RI and RI groups were statistically different than OJ and C groups ($p < 0, 05$). There was no difference between OJ+RI and RI groups histologically. The levels of urea, creatinine and bilirubin and the scores of histologic evaluations in all groups are listed in Table I.

The measurements of XOD activities were significantly elevated in OJ+RI and RI groups in 3-day post-ischemic kidneys ($p < 0,05$) and by comparison of these groups, the levels of enzyme activities in OJ+RI group were higher than in RI groups ($p < 0,05$) (Fig. 1A).

The activity of SOD was significantly decreased in both jaundiced and non-jaundiced groups in the 3-day post-ischemia ($p < 0,05$) (Fig. 1B). A similar trend was observed in GSH-Px activity ($p < 0,05$) (Fig. 1C). Comparing the groups in which the renal ischemia/reperfusion were applied, there were no differences statistically. Decreased levels of catalase activities in OJ+RI and RI groups, were not significant ($p < 0,05$) Fig. 1D). The enzyme activities of in all groups are shown in Table II.

TABLE I The levels of urea, creatinine and bilirubin in blood and the average scores of histologic findings

| Groups | No. of rats | Urea (mg%) Mean ± SD | Creatinine (mg%) Mean ± SD | Bilirubin (mg%) Mean ± SD | Scores of histology Mean ± SD |
|---|---|---|---|---|---|
| OJ+RI | (*n*=7) | 76,85 ± 12,11[1] | 1,90 ± 0,43[1] | 5,24 ± 0,95[2] | 1,45 ± 0,27[2] |
| OJ | (*n*=7) | 42,42 ± 4,92 | 0,50 ± 0,25 | 3,75 ± 0,98[2] | 0,56 ± 0,3[3] |
| RI | (*n*=7) | 60,85 ± 14,87[2] | 1,32 ± 0,45[2] | 0,45 ± 0,12 | 1,30 ± 0,20[2] |
| C | (*n*=7) | 36,42 ± 6,90 | 0,37 ± 0,18 | 0,41 ± 0,15 | 0 ± 0 |

OJ (obstructive jaundice), RI (renal ischemia/reperfusion) and C (control). All values are expressed as mean ± SD (standard deviation)
[1] $p < 0,05$ (group OJ+RI versus all other groups)
[2] $p > 0,05$ (second and third columns; group RI versus OJ and C groups)
(fourth column; jaundiced groups versus non-jaundiced groups)
(fifth column; ischemia groups versus non-ischemic groups)
[3] $p < 0,05$ (OJ group versus C group).

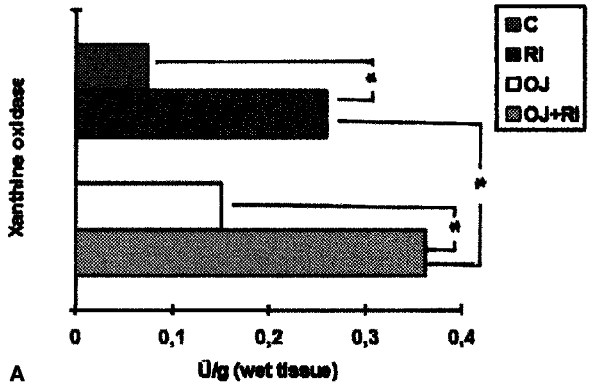

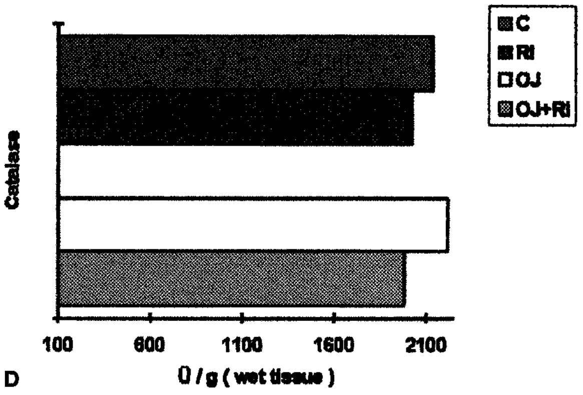

FIGURE 1 (Continued).

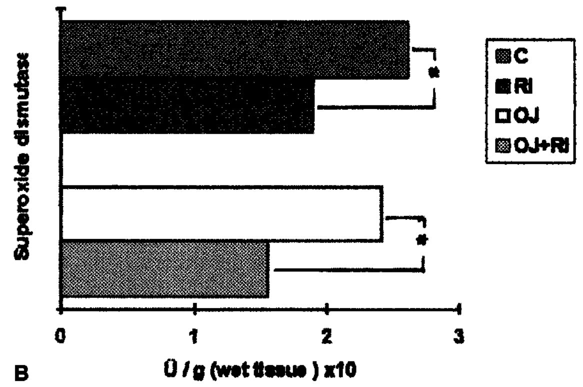

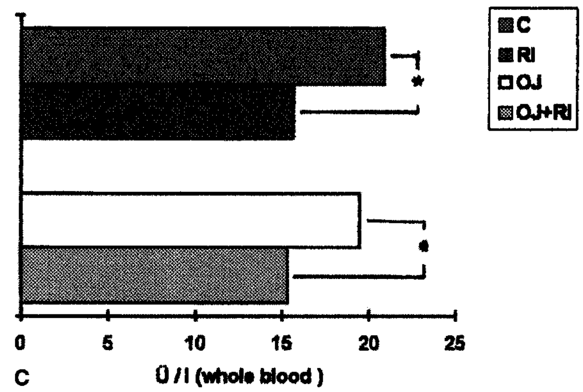

FIGURE 1 A, B, C, and D – The activities of xanthine oxidase and antioxidant enzymes are presented. OJ+RI (obstructive jaundice + renal ischemia), OJ (obstructive jaundice), RI (renal ischemia) and C (control) groups are determined in legends. * p<0,05.

### DISCUSSION

Acute renal failure (ARF) occuring in obstructive jaundice was first reported by Clairmont and von Haberer [27]. However, the ethiology of this syndrome is still unclear. Cholangitis and surgical intervention were considered to be the inducing factors. Bilirubin, bile salts, cholesterol, dissemineted intravascular coagulation (DIC), impaired cardiac performance, hypotension, hypovolemia and endotoxemia are claimed to have role in the pathophysiology [1–4], [7].

In our study, when blood urea and creatinine are taken into consideration, the renal failure was greater in OJ ± RI group when compared to RI group ($p < 0{,}05$). Histological findings of renal failure seen in both jaundiced and non-jaundiced ischemic groups did not show a significant differences, whereas when compared with the non-ischemic groups marked changes were detected ($p < 0, 05$). In OJ group which was not exposed to ischemia/reperfusion, appreciable changes in tubuler cytoplasmic vacuolisation and the loss of brush borders, expressed by the score 0,56 ± 0,3, were observed when compared to control group ($p < 0{,}05$). These observations support the idea that, the risk of renal failure is greater in obstructive jaundice.

It is reported that, ischemia and nephrotoxicity are important factors in the pathogenesis of ARF

TABLE II The levels of Xanthine oxidase and antioxidant enzyme activities

| Groups and No. of rats | | XOD (Ü/g wt) Mean ±SD | SOD (Ü/wt) × $10^3$ Mean ± SD | CAT (Ü/g wt) Mean ± SD | GSH-Px [Ü/L (blood)] ort ± SD |
|---|---|---|---|---|---|
| OJ ± RI | ($n$=7) | 0,363 ± 0,03[1] | 1,55 ± 0,35[2] | 1982,1 ± 156,5 | 15,28 ± 2,55[2] |
| OJ | ($n$=7) | 0,150 ± 0,04 | 2,41 ± 0,33 | 2217,1 ± 206,8 | 19,43 ± 1,05 |
| RI | ($n$=7) | 0,259 ± 0,01[2] | 1,89 ± 0,11[2] | 2022,8 ± 211 | 15,68 ± 2,82[2] |
| C | ($n$=7) | 0,074 ± 0,05 | 2,61 ± 0,25 | 2138,5 ± 141,7 | 20,84 ± 0,83 |

XOD (Xanthine oxidase), SOD (superoxide dismutase), CAT (catalase), GSH-Px (Glutathione peroxidase), wt (wet tissue), OJ (obstructive jaundice), RI (Renal ischemia/reperfusion), C (control). All values are expressed as mean ± SD (standard deviation)
[1]$p$ < 0,05 (OJ+RI group versus all other groups)
[2]$p$ < 0,05 (second column; RI group versus and C groups)
(third and fifth columns; Ischemia groups versus non-ischemia groups).

and their effects on renal cells can be listed as follows; I-decrease of Adenosine triphosphate (ATP) and as a consequence increase of $Ca^{++}$ in cytosol, II- loss of superoxide dismutase and superoxide radical accumulation [2, 12].

Renal ischemia results in a rapid decrease of ATP in tissue and rise in adenosine, inosine and hypoxanthine. The accumulation of hypoxanthine during renal ischemia might be related to the generation of highly reactive oxygen species, since the enzymatic conversion of hypoxanthine to xanthine by xanthine oxidase generates superoxide radical. Oxygen free radicals produce damage to the renal arteriolar endothelial cells, glomerular mesangical cells, and renal tubular epithelial cells [2, 12, 15]. Allopurinol and its metabolite oxypurinol which inhibit xanthine oxidase have a protective role against ARF occuring after renal ischemia [13, 14, 28]. A major protective mechanism against reactive oxygen metabolites is also the antioxidant enzyme cascade. It is reported that, exogenous antioxidant enzyme administration can attenuated the renal injuries in many studies [12, 13, 15].

It is suggested that, bilirubin and bile salts impair cellular metabolism and membrane transport systems. The changes in ultrastructure of glomeruler endothelial cells in Jaundice are similar to the effects of anoxia. Presumably these cells are especially sensitive to a small fall in oxygen delivery [1–3]. Decrease in renal blood flow and the redistribution of blood flow from the outer to the inner cortex is seen in bile duct ligated animal models [1, 3, 4, 9]. The profound disturbances of body-fluid compartments and extracellular and intracellular volume depletion have been detected in obstructive jaundice [10]. It is reported that, in non-jaundiced volume depleted rats, decrease in antioxidant enzyme activities has been detected [16]. This observation suggested us that the volume depletion seen in obstructive jaundice may be important in the oxidant injury of kidneys.

In obstructive jaundice, endotoxin is found in 50–75 % of patients as detected by the Limulus-Lisate assay. An increase in release of cathecolamines caused by endotoxemia is associated with a decrease in the renal blood flow. The development of disseminated intravascular coagulation (DIC) and renal fibrin deposistion are also claimed to be the cause of renal failure in obstructive jaundice. Some experimental sepsis and endotoxin studies have shown accumulation of leucocytes within glomeruli and tubules. Indeed, activated leucocytes secrete proteases and oxygen free radicals which may damage the glomerular basement membrane. Endotoxin, which may cause elevation in XOD activity and the stimulation of the lipoxygenetic pathway of arachidonic acid metabolism, also increases the secretion of oxygen free radicals [2–4, 12, 17, 18]. Moreover, it is claimed that dimethylthiourea and superoxide dismutase which are used in endotoxemia induced ARF may reduce renal demage [29]. It is suggested that, endotoxin, present in the peripheral circulation may play an important role in the development of ARF by enhancing the liberation of reactive oxygen species in jaundiced patients.

In our study, when C and OJ groups are compared with both of the ischemia/reperfusion groups in the 3-day post ischemic kidneys, the significant increase in XOD activities were observed ($p < 0{,}05$). Furthermore, the XOD activities of OJ+RI group were significantly higher than those of RI group ($p < 0{,}05$).

Changes in liver antioxidant defenses were searched for in the bile duct ligated rats and a reduction in vitamin E and selenium was observed. In addition, significant decreases in the activities of catalase, glutathione peroxidase and glutathione reductase and an increase in lipid peroxidation were found. The importance of oxidant injury was emphasized in obstructive jaundice [19]. In addition, it is claimed that a large amount of reactive oxygen species were produced by polymorphonuclear leucocytes in deeply jaundiced patients these agents may play a role in multi organ damage [19]. In a study, it was shown that the renal damage caused by gentamycin induced ROS production was more serious because of marked production of oxydants in jaundiced [20]. In another experimental study, the functional role of intrinsic antioxidant enzymes in renal oxidant injury was assessed and the reduction in enzymes activities were observed in 3-day post ischemic rats, but the elevations of activities were detected in 6-day post-ischemic rats [30]. Southard *et al.* [31] had also shown that XOD activities increased by degrees on day 3 and day 5 in kidney ischemia. On the contrary a decrease was observed in SOD activities on the same days. It been suggested that, the increase in XOD/SOD ratio may indicate oxygen derived free radical damage.

In our study, in OJ+RI and RI groups, SOD and GSH-Px (whole blood) values were significantly lower than that of control and OJ groups on the post ischemic third day ($p < 0{,}05$). Despite the lower enzyme values in jaundiced groups, the difference was not significant when compared to non-jaundiced groups. The catalase levels were not significant between the jaundiced and non-jaundiced groups.

Our results of XOD have suggested that, the generation of superoxide radical might be more pronounced in jaundiced rats. If the decrease in the SOD and GSH-Px activities should also be taken into consideration, oxidant injury may also play an important role in the development of ARF seen in obstructive jaundice. The importance of free radical injury in obstructive jaundice clearly need further investigations.

### *References*

[1] Coratelli, P. and Passavanti, G. (1990). Pathophysiology of Renal Failure in Obstructive Jaundice. *Miner electrolyte Metab.*, **16**, 61–5.

[2] Allison, M. E. M. (1990). The kidney and the liver. Prè- and postoperative factors. (Ed.) Blumgart, L.G. Surgery of Liver and Biliary Tract. First edition, Vol. **1**, Churchill Livingstone, London p. 405–421.

[3] Wait, R. B. and Kahng, K. U. (1989). Renal failure Complicating Obstructive Jaundice. *Am. J. Surg.*, **157**, 256–63.

[4] Fletcher, M. S., Westwich, J. and Kakkar, V. V. (1982). Endotoxin, prostaglandins and renal fibrin deposition in obstructive jaundice. *Br. J. Surg.*, **89**, 625–29.

[5] Cioffi, W. G., DeMeules, J. E., Kahng, K. U. and Wait, R. B. (1986). Renal vascular reactivity in jaundice. *Surgery*, **100**, 356–62.

[6] O'Neil, P. A., Wait, R. B. and Kahng, K. U. (1990). Obstructive jaundice and renal failure in the rat: The role of renal prostaglandins and the renin-angiotensin system. *Surgery*, **108**, 356–62.

[7] Thompson, J. N., Edwards, W. H., Winerals, C. G., Blenkham, J. I., Benjamin, I. S. and Blumgart, L. H. (1987). Renal impairment following biliary tract surgery. *Br. J. Surg.*, **74**, 843–47.

[8] Pain, J. A., Cahill, C. J., Gilbert, J. M., Johnson, C. D., Trapnell, J. E. and Bailey, M. E. (1991). Prevention of postoperative renal dysfunction in patients with obstructive jaundice: a multicentre study of bile salts and lactulose. *Br. J. Surg.*, **78**, 467–69.

[9] Tobimatsu, M., Ueda, Y., Saito, S., Tsumagari, T. and Konomi, K. (1988). Effects of a Stable Proctacyclin Analog on Experimental Ischemic Acute Renal Failure. *Ann. Surg.*, **208**, 65–70.

[10] Gubern, J. M., Sancho, J. J., Simo, J. and Sitges-Serra, A. (1988). A randomize trial on the effect of mannitol on postoperative renal function in patients with obstructive jaundice. *Surgery*, **103**, 39–44.

[11] Parks, R. W., Diamond, T., McCrory, D. C., Johnston, G. W. and Rowlands, B. J. Prospective study of post-operative renal function in obstructive jaundice and the effect of perioperative dopamine. *Br. J. Surg.*, **81**, 437–9.
[12] Paller, M. S., Hoidal, J. R. and Ferris, T. F. (1984). Oxygen Free Radicals in Ischemic Acute Renal Failure in the Rat. *J. Clin. Invest.*, **74**, 1156–64.
[13] Burke, T. J. and Schrier, R. W. (1993). Pathophysiology of Cell Ischemia. (Eds.) Schrier, R. W., Gottschalk, C. W. Diseases of the Kidney. Fifth ed. Vol. **II**, Little, Brown and Company, Boston, p. 1257–86.
[14] Schrier, R. W. and Burke, T. J. (1994). New aspects in pathogenesis of acute renal failure. *Nephrol. Dial. Transplant*, **9**(Suppl. 4), 9–14.
[15] Yoshioka, T. and Ichikawa, I. (1994). Cellular defence mechanism against ischaemic and toxi injury. *Nephrol Dial Transplant*, **9**(Suppl. 4), 34–36.
[16] Yoshioka, T., Fogo, A. and Beckman, J. K. (1992). Reduced activity of antioxidant enzymes underlies contrast media-induced renal injury in volume depletion. *Kidney. Int.*, **41**, 1008–1015.
[17] Klahr, S. (1994). Role of arachidonic acid metabolites in acute renal failure and sepsis. *Nephrol. Dial. Transplant*, **9**, (Suppl. 4), 52–6.
[18] Flohe, L. and Giertz, H. (1987). Endotoxins, H. arachidonic acid, and superoxide formation. *Rev. Infect. Dis.*, **9**, (Suppl. 5), 553–61.
[19] Ohshio, G., Miyachi, Y., Kudo, H., Niwa, Y., Manabe, T. and Tobe, T. (1988). Effects of sera from patients with obstructive jaundice on the generation of oxygen intermediates by normal polymorphonulear leukocytes. *Liver*, **8**, 366–71.
[20] Tajiri, K., Miyakawa, H., Marumo, F. and Sato, C. (1995). Increased renal susceptibility to gentamicin in the rat with obstructive jaundice : Role of Lipid peroxidation. *Dig. Dis. Sci.*, **40**, 1060–60.
[21] Singh, S., Shackleton, G., Ah-sing, E., Chakraborty, J. and Bailey, M. E. (1992). Antioxidant Defenses in the Bile Duct-Ligated Rat. *Gastroenterolgy*, **103**, 1625–29.
[22] Hashimoto , S. (1974). A new Spectrophotometric assay method of xanthine oxidase in crude tissue homogenates. *Annal. Biochem.*, **62**, 426–35.
[23] Sun, Y. (1988). A simple Method for Clinical Assay of superoxide Dismutase. *Clin. Chem.*, **34**, 497–500.
[24] Beers, R. F. (1952). A Spectrophotometric method for Measuring the Breakdown of Hydrogen Peroxide by Catalase. *I. Biol. Chem.*, **195**, 133–40.
[25] Paglia, D. E. and Valentine, W. N. (1967). Studies on the quantitative and qualitative characterization of erythrocyte glutathione peroxidase. *J. Lab.Clin. Med.*, **70**, 58–69.
[26] Dawson-Saunders, B. and Trapp, R. G. (1990). Basic and Clinical Biostatistics. First ed, Appleton-Lange, USA.
[27] Clairmont, P. and Von Haberer, H. M. (1911). Über anurie nach gallenstein operatonen. *Grenzgeb. Med. Chir.*, **22**, 159–72.
[28] Wasko, K. A., DeWall, R. A. and Riley, A. M. (1972). Effect of allopurinol in ischemia. *Surgery*, **71**, 787– 90.
[29] Zurovsky, Y. and Gispaan, I. (1995). Antioxidants attenuate endotoxin-induced acute renal failure in rats. *Am. J. Kidney. Dis.*, **25**, 51–7.
[30] Yoshioka, T., Bills, T., Moore-Jarrett, T., Greene, H. L., Burr, I. M. and Ichikawa, I. (1990). Role of intrinsic antioxidant enzymes in renal oxidant injury. *Kidney. Int.*, **38**, 282–8.
[31] Southard, J. H., Marsh, D. C., McAnulty, J. F. and Belzer, F. O. (1987). Oxygen – derived free radical damage in organ preservation : Activity of superoxide dismutase and xantine oxidase, *Surgery*, **101**, 566–70.

### COMMENTARY

Renal failure in patients with obstructive jaundice typically occurs when cholangitis is present or in the postoperative phase. Although historical data suggests that there is a high incidence, recent clinical experience suggests that it is now much less of a problem. Renal failure in patients presenting with cholangitis may be prevented by a adequate resuscitation combined with early endoscopic drainage. Patients undergoing surgery for obstructive jaundice frequently now undergo preoperative endoscopic drainage, and this with full hydration over the perioperative period are likely to be important factors in the protection of renal function.

Despite its reducing incidence renal failure when it does occur is associated with a high mortality. The mechanism accounting for the renal impairment remains obscure. The findings of this paper suggest that oxygen free radicals are important mediators in the acute tubular necrosis. A word of caution should be raised though because this study used an ischaemic renal reperfusion model in the presence of obstructive jaundice a situation which may not be analogous to the situation in a jaundiced patient even one undergoing surgery. Nevertheless if oxygen free radicals do have an important pathophysiological role then new therapeutic strategies may be developed.

Mr. J. A. Pain
Department of Surgery
Poole General Hospital
Longfleet Road
Poole
Dorset BH15 2JB
UK

